# Coil Combination of Multichannel Single Voxel Magnetic Resonance Spectroscopy with Repeatedly Sampled In Vivo Data

**DOI:** 10.3390/molecules26133896

**Published:** 2021-06-25

**Authors:** Wanqi Hu, Huiting Liu, Dicheng Chen, Tianyu Qiu, Hongwei Sun, Chunyan Xiong, Jianzhong Lin, Di Guo, Hao Chen, Xiaobo Qu

**Affiliations:** 1 Department of Electronic Science, Fujian Provincial Key Laboratory of Plasma and Magnetic Resonance, National Institute for Data Science in Health and Medicine, Xiamen University, Xiamen 361005, China; wanqihu@stu.xmu.edu.cn (W.H.); madkm@163.com (H.L.); dcchen@stu.xmu.edu.cn (D.C.); tianyuqiu@stu.xmu.edu.cn (T.Q.); xiongchunyan918@126.com (C.X.); 2 United Imaging Research Institute of Intelligent Imaging, Beijing 100101, China; hongwei.sun@cri-united-imaging.com; 3 Magnetic Resonance Center, Zhongshan Hospital, Xiamen University, Xiamen 361004, China; xmzshljz123@163.com; 4 School of Computer and Information Engineering, Xiamen University of Technology, Xiamen 361024, China; guodi@xmut.edu.cn; 5 School of Biomedical Engineering, Shanghai Jiao Tong University, Shanghai 200030, China

**Keywords:** biomolecular NMR, magnetic resonance spectroscopy, SNR, coil combination, SVD

## Abstract

Magnetic resonance spectroscopy (MRS), as a noninvasive method for molecular structure determination and metabolite detection, has grown into a significant tool in clinical applications. However, the relatively low signal-to-noise ratio (SNR) limits its further development. Although the multichannel coil and repeated sampling are commonly used to alleviate this problem, there is still potential room for promotion. One possible improvement way is combining these two acquisition methods so that the complementary of them can be well utilized. In this paper, a novel coil-combination method, average smoothing singular value decomposition, is proposed to further improve the SNR by introducing repeatedly sampled signals into multichannel coil combination. Specifically, the sensitivity matrix of each sampling was pretreated by whitened singular value decomposition (WSVD), then the smoothing was performed along the repeated samplings’ dimension. By comparing with three existing popular methods, Brown, WSVD, and generalized least squares, the proposed method showed better performance in one phantom and 20 in vivo spectra.

## 1. Introduction

Magnetic resonance spectroscopy (MRS), as a useful tool for determining the in vivo molecular compositions, has achieved impressive success over the past two decades. One of the main clinical application of MRS is to quantify the concentration of metabolites, especially for the analysis of the brain neurochemistry changes that are associated with some brain diseases like tumor [1,2], Alzheimer disease [3,4], Parkinson [5], and stroke [6]. However, due to the low concentration of some metabolites and the relatively low signal-to-noise ratio (SNR), further quantification and analysis of metabolites is difficult to be promoted for the brain spectrum [7,8].

**Figure 1:**
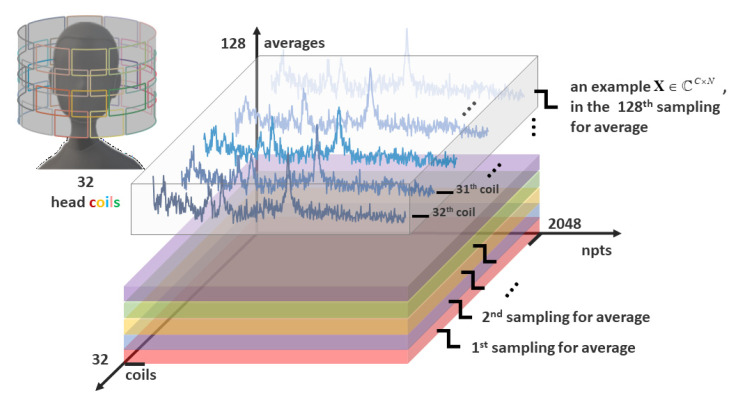
An illustration of the array coil acquisition with repeatedly sampled, three-dimensions data acquisition $\tilde{X}\in {\mathbb{C}}^{C\times A\times N}$ (coils (*C*), averages (*A*), npts (*N*)).

There are two different conventional methods for improving the SNR of MRS. One is to average signals obtained from the repeated collections, regarded as the averages’ dimension *A* (size 128 in Figure 1). The other is to receive multichannel spectra from phase arrays and combine them by signal processing, regarded as the coils’ dimension *C* (size 32 in Figure 1). Take the number of sampling points of MRS as npts dimension *N* (size 2048 in Figure 1), then the whole three-dimensions’ MRS acquisition is shown in Figure 1. The multichannel coil acquisition, which was first proposed by Roemer et al. [9], simultaneously acquires data from multiple, closely overlapping magnetic resonance receiving arrays in the region of interest and has been applied in MRS and magnetic resonance imaging (MRI) [10,11,12]. Based on Roemer theory, several coil-combination signal processing methods have been proposed for maximizing the SNR. These methods form a linear combination of spectra with weights (sensitivities matrix) that provide constructive addition of the signals and give higher emphasis to coils with higher signal [13]. An easy evaluation of the weights is taking advantage of characteristics of the signal itself, like the amplitude of metabolite peak [14], unsuppressed water peak [15], or the first point of its time-domain signal [15] as the weighting coefficient. However, the above methods ignore the correlation of the noises among coils in practice. Hence, Rodgers and Robson [16] proposed a whitened singular value decomposition (WSVD) method, aiming to reduce the noise correlation using whitening before the singular value decomposition process. Another method, named generalized least squares (GLS) [17], which solves the inverse problem of signal recovery by using generalized least squares, makes the coefficient of variation of the peak smaller and provides a more reliable pretreatment for the quantification of metabolites. Nevertheless, the improvement of SNR is still not satisfying enough. One possible promotion method is utilizing the information of two acquisition ways simultaneously.

In this paper, we proposed a WSVD-based advanced coil-combination method, which is named as average smoothing singular value decomposition (ASSVD), to further utilize the similarity between each repeated samplings within one coil. This similarity is achieved by smoothing the coil sensitivity map along the npts dimension N and averages’ dimension A with a two-dimensions’ averaging convolution kernel.

## 2. Materials and Methods

### 2.1. Materials

All the MRS data were collected on a 3T United Imaging (Shanghai, China) scanner with 32-channel phase array head coils. The point resolved spectroscopy (PRESS) [18] sequence was performed, which consisted of three slice-selective RF pulses (90°-180°-180°). The experimental parameters were as follows: TR/TE = 2000/30 ms, voxel size = 20 mm × 20 mm × 20 mm, number of points = 2048, and spectral bandwidth = 2000 Hz. Water suppression was applied using the WET [19], while a water-unsuppressed spectrum was also collected as the reference signal for the absolute concentration quantification and phase correction. For each spectrum, 128 repeated samplings took about 4 min, 21 s. Finally, the MRS raw data acquired from the machine before coils combination were three-dimensional with *C* = 32, *A* = 128, and *N* = 2048 and there was no additional preprocessing.

#### 2.1.1. Phantom Experiments

**Figure 2:**
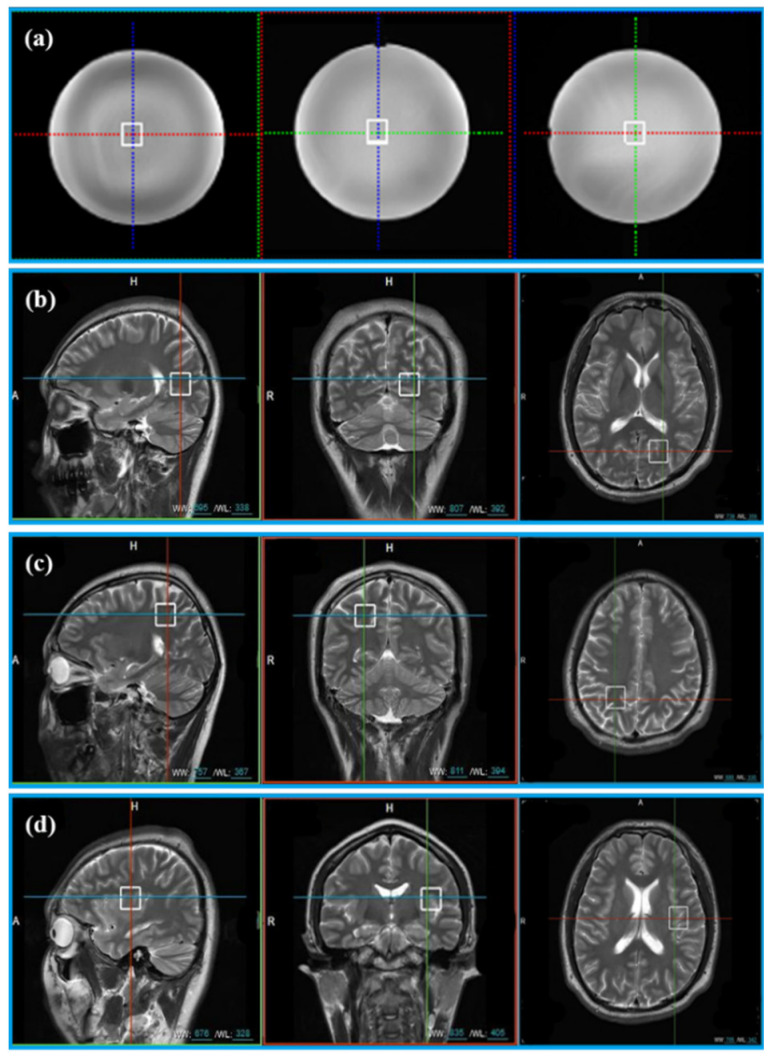
The location of voxels acquired from phantom and 11 healthy volunteers. (**a**) Voxel location of the phantom; (**b**) voxel LA, (**c**) voxel LB, and (**d**) voxel LC are the voxel locations of 11 healthy subjects.

The General Electric golden standard phantom sphere was used for phantom experiments. Voxel locations of the sphere are shown in Figure 2a. The simulated correlation noise between coils was added to phantom experiments with different noise levels [13,16,17]. The simulated noise of each repeated sampling was independently generated according to the in vivo spectrum as follows.

For the noise **E**, which is often unknown in practical problems, one accepted way is to regard a range of spectrum, such as the 0.4–1.0 ppm range of ^1^H MRS, as the noise-only measurement region [13]. Then, replace the realistic **E** with the estimated $\hat{E}$ obtained from the region. The estimated noise covariance matrix $\hat{\Psi }$ can be expressed as Formula (1) [13,17]:

(1) $$\hat{\Psi }=\hat{E}{\hat{E}}^{H}$$

The experimental correlation noise matrix $\hat{\Psi }$ was independently measured from each repeated sampling of in vivo spectrum in the noise-only region. Then, we generated a white Gaussian noise matrix ${Ε}_{\mathrm{Gaussian}}\in {\mathbb{C}}^{C\times N}$, whose real and imaginary entries belong to the standard normal distribution. The correlation noise ${Ε}_{\mathrm{corr}}$ was generated as:

(2) $$\begin{array}{c} \hat{\Psi }=LL^{H}\\ {Ε}_{\mathrm{corr}}=L^{H}{Ε}_{\mathrm{Gaussian}} \end{array}$$

where ${}^{H}$ denotes the conjugate transpose operation, L denotes the Cholesky decomposition of $\hat{\Psi }$, and ${Ε}_{\mathrm{corr}}$ denotes the correlation noise called unit (1×) noise level. The phantom data added to the simulated correlated noise in the time domain was expressed as:

(3) $$X_{q-\mathrm{noise}}=X+q{Ε}_{\mathrm{corr}},\;q=1,2,\ldots ,7$$

#### 2.1.2. In Vivo Experiments

In vivo data were acquired from 11 healthy volunteers with the approval of the Institutional Review Board of Shanghai Jiao Tong University. Informed consent was obtained from all volunteers before the study began. Three healthy volunteers were scanned at three different voxel locations (Location A (LA), Location B (LB), and Location C (LC)) in Figure 2b–d. Five healthy subjects were scanned at the LA only and other three healthy subjects were scanned at LB and LC. Finally, totally, 20 MRS were acquired for validation (see Table 1 for details).

### 2.2. Methods

In practice, the collected MRS data $\tilde{X}\in {\mathbb{C}}^{C\times A\times N}$ were multi-dimensional, as shown in Figure 1, where the number of coils, averages, and signal points are denoted as *C*, *A*, and *N*, respectively.

According to Roemer’s multichannel phase array receive coils theory [9], for *C* coils phase array scanner, the received spectrum with *N* points acquired from the *c*th coil can be expressed as:

(4) $$X_{c}(n)=s_{c}m(n)+E_{c}(n),\;n=1,2,\ldots ,N,\;c=1,2,\ldots ,C,$$

where $s_{c}$ represents the sensitivity and phase shift of coil *c*, m(n) is the *n*th point of the ideal high SNR combination spectrum, and $E_{c}(n)$ could be considered as Gaussian white noise, which mainly arises from the thermal fluctuations within the subject [16]. These values depend on the location of the exciting voxel relative to the coil, the array design, the geometry and dielectric properties of the sample, the gain of the receiving coil, etc. [16]. The coils’ combination can be modeled according to the corresponding matrix form of Formula (4) as:

(5) $$X=sm^{T}+E,$$

where ${}^{T}$ denotes the transpose operation and $X\in {\mathbb{C}}^{C\times N}$ and $E\in {\mathbb{C}}^{C\times N}$ are the acquisition signal matrix (the example shown in Figure 1) and the correlation noise matrix, respectively. The $s\in {\mathbb{C}}^{C\times 1}$ stands for the sensitivity vector of the coil array and $m\in {\mathbb{C}}^{N\times 1}$ represents the ideal high SNR coil-combined spectrum.

In the next two subsections, we briefly review three typical combined methods, Brown [15], GLS [17], and WSVD [16]. Then, we propose a new method, average smoothing singular value decomposition (ASSVD) in Section 2.2.3.

#### 2.2.1. Brown and GLS

As described in the literature [15], the combined result ${\hat{m}}_{\mathrm{Brown}}$ of Brown’s method was obtained by weighting each coil $x_{c}$ with the first point $X_{c}(1)$ of the MRS time-domain signal:

(6) $$\;{\hat{m}}_{\mathrm{Brown}}=\sum_{c=1}^{C}\left(X_{c}(1\right)\times x_{c})$$

GLS algorithm solves the general model (5) through the generalized least square method [17]. If the noise **E** is known to Gaussian normal distribution with the mean of zero, the best linear unbiased estimation ${\hat{m}}^{}{}_{\mathrm{GLS}}$ of the problem (5) is expressed as follows [17]:

(7) $${\hat{m}}^{}{}_{\mathrm{GLS}}={\left({\left(s^{H}{\hat{\Psi }}^{-1}s\right)}^{-1}s^{H}{\hat{\Psi }}^{-1}X\right)}^{T}$$

Here, the sensitivity vector **s** consists of the peak value of NAA at 2.0 ppm from each coil, $\hat{\Psi }$ is the noise covariance matrix, which could be calculated by Formula (1), and ${}^{-1}$ denotes the inverse operation.

#### 2.2.2. WSVD

The WSVD is a modified SVD-based method, which considers that the noises across coil arrays are correlated [16]. Therefore, we briefly went through SVD before WSVD.

In model (5), to solve the sensitivity vector s and the ideal coil-combined spectrum m, the acquired MRS matrix $X\in {\mathbb{C}}^{C\times N}$ was used for SVD decomposition [16]:

(8) $$X=U\Sigma V^{H},$$

where $U\in {\mathbb{C}}^{C\times C},V\in {\mathbb{C}}^{N\times N}$ are the unitary matrices and Σ represents the diagonal matrix composed of descending singular values. The left and right singular vectors corresponding to the maximum singular value $\Sigma_{11}$ are $u_{C1}$ and $v_{N1}$, respectively.

The coil-combined spectrum ${\hat{m}}_{\mathrm{SVD}}$ can be solved by the following formula [16,20] under the assumption that the max contribution or the principal component of Σ is the solution:

(9) $$\begin{array}{c} {\hat{m}}_{\mathrm{SVD}}={\left(\Sigma_{11}v_{N1}^{H}\xi \right)}^{T}\\ \hat{s}=\frac{u_{C1}}{\xi }, \end{array}$$

where $\hat{s}$ is the estimated sensitivity matrix and ξ is the free amplitude/phase of each voxel.

The difference between WSVD and SVD is that WSVD does the noise whitening for decorrelation before the SVD operation. The whitened process is expressed as below:

(10) $$X_{\mathrm{Whitened}}=WX,$$

where matrices $X_{\mathrm{Whitened}}$ and X denote the whitening data matrix and the data acquisition matrix, respectively. The matrix $W\in {\mathbb{C}}^{C\times C}$ is the whitening weight matrix constructed according to the eigen decomposition of the noise covariance matrix $\hat{\Psi }$ as below:

(11) $$\begin{array}{c} \hat{\Psi }=GDG^{H}\\ W=D^{-1/2}G^{H}, \end{array}$$

where the complex matrix G consists of eigenvectors of $\hat{\Psi }$ and the corresponding eigenvalues are arranged at the diagonal matrix D.

**Figure 3:**
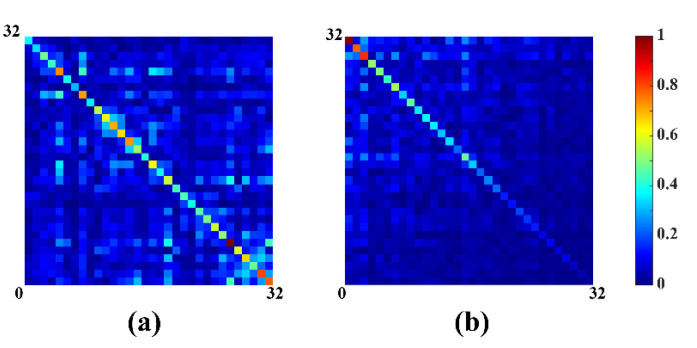
Noise correlation matrix of 32 coils before and after whitened. (**a**) Before whitened; (**b**) after whitened.

Results in Figure 3 show the correlation matrix of noise before (Figure 3a) and after whitened (Figure 3b), indicating the correlation matrix after whitened is closer to the identity matrix. Finally, substitute $X_{\mathrm{Whitened}}$ for X in (8) and the ideal coil-combined spectrum can be expressed as:

(12) $$\begin{array}{c} X_{\mathrm{Whitend}}=\tilde{U}\tilde{\Sigma }{\tilde{V}}^{H}\\ {\hat{m}}_{\mathrm{WSVD}}={\left(W^{-1}{\tilde{\Sigma }}_{11}{\tilde{v}}_{N1}^{H}\xi \right)}^{T}, \end{array}$$

Because $D^{-1/2}$ and $G^{H}$ are usually full-rank matrices, the inverse whitening operation $W^{-1}$ always can be calculated.

#### 2.2.3. The Proposed Method

Based on the WSVD, which de-correlates the noise by signal whitening, we proposed a multicoil channel-combination method with the repeated samplings, ASSVD, which extracts the information among the repeated samplings through the convolution to gain a higher SNR. The advantages of WSVD are absorbed into the proposed method. In the meanwhile, ASSVD takes the relationship between repeated samplings into consideration, making the sensitivity matrix between each repeated sampling smoother. The model and its solution process are shown as follows.

The repeatedly sampled data acquisition matrix $\tilde{X}$ (shown in Figure 1) can be reshaped to a complex vector $\tilde{x}\in {\mathbb{C}}^{CAN\times 1}$. For the *a*th sampling and the *c*th coil, the relationship among the measurement data $x_{c,a}\in {\mathbb{C}}^{N\times 1}$, the sensitivity $s_{c,a}\in {\mathbb{C}}^{N\times 1}$, and the pre-combined signal ${\hat{m}}_{a-\mathrm{WSVD}}\in {\mathbb{C}}^{N\times 1}$ obtained from WSVD can be stated as below:

(13) $$x_{c,a}=s_{c,a}^{}\cdot \;{\hat{m}}_{a-\mathrm{WSVD}},\;a=1,2,\ldots ,A,\;c=1,2,\ldots ,C$$

where · donates the vector dot product.

**Figure 4:**
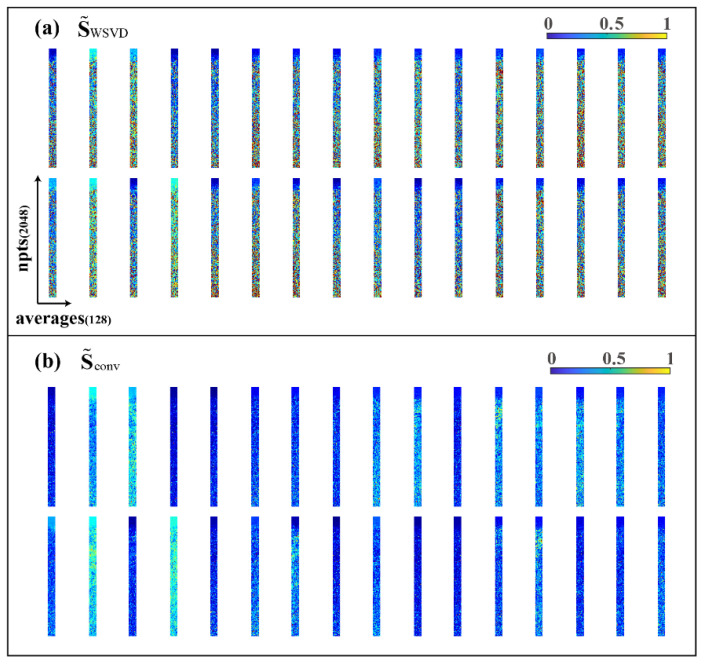
Visualization of 32 coil sensitivity matrices. (**a**) Sensitivity matrix obtained from WSVD; (**b**) sensitivity matrix after convolution smoothing (convolution kernel size *k* is 7).

Presume that the sensitivity of each coil is stable between two arbitrary repeated samplings by the idea that the magnetic field distribution of its space relative to the coil array is constant in the same voxel. Under the assumption, the sensitivity $s_{c,a}$ is arranged into a new complex sensitivity matrix ${\tilde{S}}_{\mathrm{WSVD}}$ as below:

(14) $${\tilde{S}}_{\mathrm{WSVD}}=\left[\begin{array}{ccccccc} s_{1,1}(1) & & & & & & \\ & \cdots & & & & & \\ & & s_{1,1}(n) & & & & \\ & & & \cdots & & & \\ & & & & s_{1,a}(1) & & \\ & & & & & \cdots & \\ & & & & & & s_{1,a}(n)\\ & & & \vdots & & & \\ s_{c,1}(1) & & & & & & \\ & \cdots & & & & & \\ & & s_{c,1}(n) & & & & \\ & & & \cdots & & & \\ & & & & s_{c,a}(1) & & \\ & & & & & \cdots & \\ & & & & & & s_{c,a}(n) \end{array}\right]\in {\mathbb{C}}^{CAN\times AN},$$

where $s_{c,a}(n)$ is the sensitivity value of point n=1,2,…,N of $s_{c,a}$ and all the ungiven blank positions are zero value. Therefore, by reshaping the non-zero parts of the matrix ${\tilde{S}}_{\mathrm{WSVD}}$ in the form of C×A×N (as shown in Figure 4a), the sensitivity matrix of each coil should be relatively smooth on the plane formed by the repeatedly sampled dimension.

To obtain a smoother sensitivity matrix, as expected, we introduced SENSE [10], an MRI multicoil-combination method, and did the two-dimensional convolution smoothing ${\tilde{S}}_{\mathrm{WSVD}}$ between each sampling by using average filtering convolution kernels φ(k) with the size of k×k. The new smooth sensitivity matrix ${\tilde{S}}_{\mathrm{conv}}$ (the non-zero part after being reshaped, as shown in Figure 4b) can be expressed as:

(15) $${\tilde{S}}_{\mathrm{conv}}={\tilde{S}}_{\mathrm{WSVD}}*\varphi (k)$$

where ∗ represents the convolution operation.

According to ${\tilde{S}}_{\mathrm{conv}}$ and $\tilde{x}$, we can obtain the idea of repeatedly sampled coil-combination ${\tilde{m}}_{\mathrm{ASSVD}}\in {\mathbb{C}}^{AN\times 1}$ through solving the model (16):

(16) $$\tilde{x}={\tilde{S}}_{\mathrm{conv}}{\tilde{m}}_{\mathrm{ASSVD}}$$

## 3. Results

### 3.1. The Evaluation Criteria

We compared ASSVD with three typical coil-combination methods: Brown [15], GLS [16], and WSVD [17]. The resultant combined spectra were evaluated by the SNR, which is defined as [21]:

(17) $$\mathrm{SNR}=\frac{\max(x-x_{\mathrm{baseline}})}{2\sqrt{\frac{1}{n}{\left\|x-x_{\mathrm{fitted}}\right\|}_{2}^{2}}},$$

where x and $x_{\mathrm{baseline}}$ denote the coil-combined spectrum and the spectral baseline, respectively. The subtractions of the fitted spectrum $x_{\mathrm{fitted}}$ by LCModel [21] and the spectrum x are the fitting residuals.

In addition, we also considered the LCModel metabolites’ quantification results in the phantom experiment. The metabolites involved and their abbreviations are listed as follows: Choline (Cho), Creatine (Cr), Glutamine (Gln), Glutamate (Glu), Glycerophosphosphocholine (GPC), L-Lactate (Lac), *myo*-Inositol (mI/Ins), *N*-acetylaspartate (NAA), *N*-acetylaspartylglutamate (NAAG), Phosphocholine (PCh), and Phosphocreatine (PCr). To better evaluate the SNR and quantify the coil-combined MRS [22], we utilized a 17-metabolites basis set, which is commonly used for brain MRS in LCModel analysis, and presented the sum concentration of similar spectra rather than single one, i.e., NAA + NAAG instead of NAA, Cr + PCr instead of Cr, GPC + PCh instead of Cho, and Glu + Gln instead of Glu. In all experiments, eddy-current correction and phase correction were automatically done by LCModel and analysis results were presented in the spectrum range of 0.2–4.0 ppm.

### 3.2. Phantom Experiment

**Figure 5:**
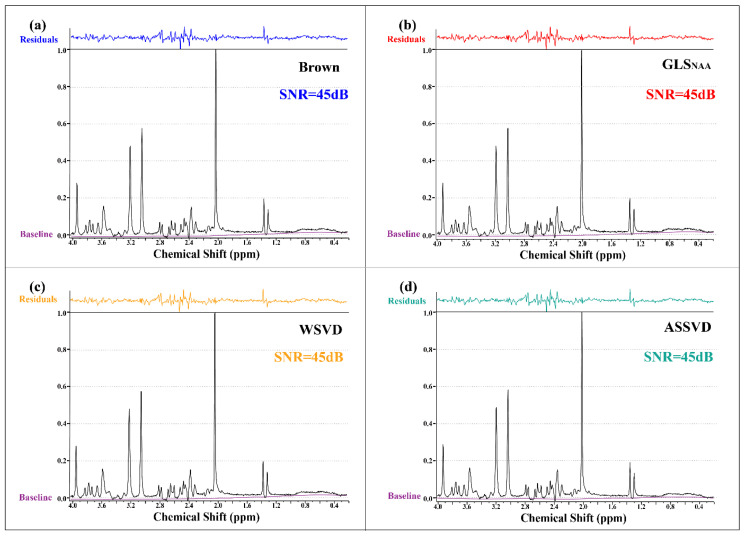
Phantom MRS coil-combined results. (**a**) Brown, (**b**) GLS using NAA peak as the reference, (**c**) WSVD, and (**d**) the proposed method ASSVD. The black and purple lines represent coil-combined MRS x and the spectral baseline $x_{\mathrm{baseline}}$ estimated by LCModel, respectively. Additionally, the fitting residuals shown at the top were calculated by $x-x_{\mathrm{fitted}}$.

The SNR results in Figure 5 show that all coil-combination methods could achieve the comparative SNR level (45 dB) in the phantom experiment. The high SNR could be attributed to the high quality of the phantom spectrum, which was caused by the richer concentration of metabolites and the absence of experimental interferences compared with in vivo MRS. It is worth noting that the significant residuals were in the range of 2.2–2.8 ppm in all methods, which was more likely to be caused by the fitting.

Results in Table 2 show the absolute quantification concentration of metabolites after coil combination under the same water reference spectrum. For ensuring the spectrum quantification was of comparative significance, we only presented the results by WSVD and ASSVD, which owned the same scale in water-referenced quantification by LCModel. Compared with WSVD, the proposed method provided the spectrum whose quantified concentrations were almost closer to the reference values and acquired a lower relative error. In addition, the overall quantification concentration of ASSVD was higher than that of WSVD, which also indicated to some extent that ASSVD could better maintain the peak intensity.

### 3.3. In Vivo Experiment

**Figure 6:**
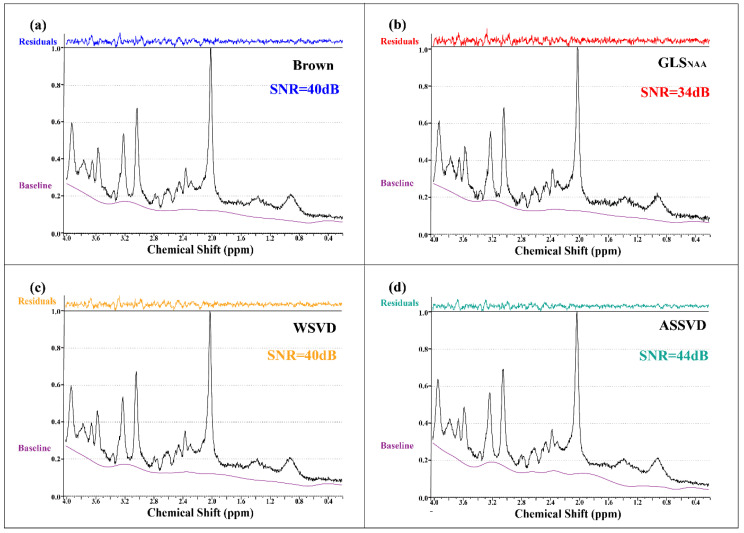
In vivo MRS coil-combined results. (**a**) Brown, (**b**) GLS using NAA peak as the reference, (**c**) WSVD, and (**d**) the proposed method ASSVD. The black and purple lines represent coil-combined MRS x and the baseline $x_{\mathrm{baseline}}$ estimated by LCModel, respectively. Additionally, the residuals shown at the top were calculated by $x-x_{\mathrm{fitted}}$.

Coil-combined in vivo spectra with four methods and the fitting residuals by LCModel are shown in Figure 6, verifying that ASSVD had a supreme SNR improvement compared with Brown and WSVD, from 40 dB to 44 dB. Additionally, in the 1.4–2.0 ppm segments, the proposed ASSVD obviously reduced noises compared with other methods, and in the 2.8–3.0 ppm and 0.4–0.6 ppm segments, the resultant spectrum also had less noise. This promotion benefited from ASSVD not only taking advantage of the multicoil acquisition but also integrating the information between repeated samplings for maximizing the SNR. Therefore, ASSVD is expectedly suitable for MRS, which was acquired with repeated samplings in routines and has a great application prospect.

## 4. Discussions

### 4.1. Influence of the Kernel Size

The only parameter of the proposed ASSVD was the size of the convolution kernel that determined the smoothing intensity of the sensitivity matrix. Table 3 presents the SNR comparison of the ASSVD method with different convolutional kernel sizes of 3, 5, 7, and 9 for the 20 in vivo spectra. For most spectra, the optimal SNR could be obtained when the convolution kernel was 7 in our data. If the kernel size was small, like 3 or 5, SNR got worse than the results of WSVD (P7-LA and P9-LB). On the other hand, a too-large kernel, whose size exceeded the optimal value, would reduce the SNR to a certain extent (P2-LC and P8-LA). Further, we applied the small size samples’ (20 MRS < 30) one-sided *t*-test [23], which is used to determine if the means of two sets of data are significantly different from each other, to demonstrate that our proposed ASSVD had significant SNR improvement with WSVD. The results are also shown in Table 3 and indicated ASSVD had a statistically significant SNR improvement (*p* value < 0.01) with WSVD in the case of Kernel Size ≥5.

To our best knowledge, there did not seem to be any linear relationship between the size of the convolution kernel and the SNR of the spectrum itself. How to choose the optimal size still remains for discussions in the future. Introducing the prior information such as Low-Rank Hankel [24,25,26] features or new technologies such as deep learning [27] to determine this parameter are both potential solutions.

### 4.2. Influence of Noise Level on the Combination Methods

To further investigate the SNR promotion of the proposed method in the strong noisy scenario, we manually added different levels of correlation noise to the phantom spectrum. The correlation noise was simulated by the noise matrix obtained from in vivo spectrum, which was denoted as unit (1×) noise. (Details of noise simulation are explained in Section 2.1.1). For ASSVD results, the size of the convolution kernel had no apparent impact on the performance and, thus, we merely presented the result when it was chosen as 7.

**Figure 7:**
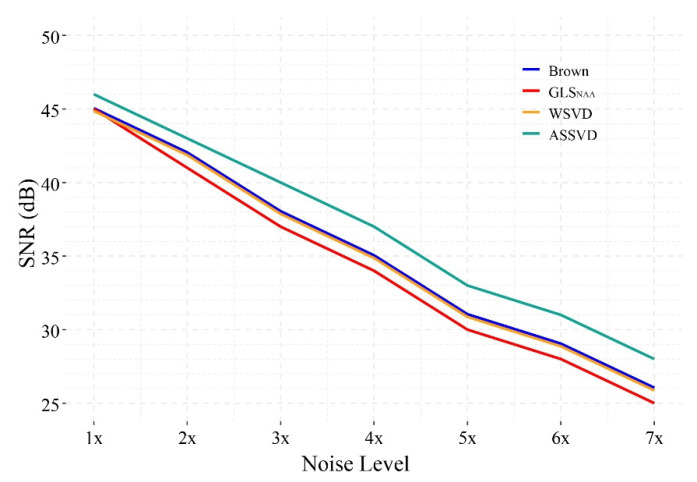
The SNR of different coil-combination results under 1×–7× simulated correlation noise.

Figure 7 shows the SNR performance of different combination methods under 1×–7× noise levels. The result indicates that, under seven different noise levels, the proposed ASSVD always achieved the highest SNR among the four methods, while WSVD and Brown method always performed comparably, but slightly better than GLS_NAA_. In the case of low noise (1× noise), the combined spectra of the three compared methods had similar SNR. With the increase of the noise level, the gaps between ASSVD and the other three methods tended to expand, illustrating that the advantage of ASSVD can be more obvious under the strong noise. In summary, ASSVD showed superiority in the presence of various levels of noise in our test, implying its wide range of application scenarios.

## 5. Conclusions

We presented a new MRS multichannel coil-combination method, ASSVD, which performed combining while taking the information of repeated sampling into consideration by a smoothing operation for promotion SNR in combined spectra. In addition, ASSVD owned merely one parameter, the size of the smooth convolution kernel, offering convenience for users. The combination results on phantom and in vivo data showed that ASSVD is capable of achieving better SNR than existing typical methods, even in the scene of strong noise. In the future, we will focus on exploring the selection of convolution kernel size so that ASSVD can automatically provide better coil-combination spectra.

## Figures and Tables

**Table 1:** The scanning single-voxel location list of each volunteer.

Person	1	2	3	4	5	6	7	8	9	10	11
Location											
LA	√	√	√	√	√	√	√	√	×	×	×
LB	√	√	√	×	×	×	×	×	√	√	√
LC	√	√	√	×	×	×	×	×	√	√	√

The √ and × indicate the data that were acquired and not acquired, respectively. To illustrate, use P2-LA to represent the spectra scanned at location A from person 2.

**Table 2:** Absolute quantification concentration (AQC) (mmol/L) of the phantom experiment.

Metabolites	Reference	Coil-Combination Methods	
		WSVD (RE)	ASSVD (RE)
**NAA** **+ NAAG**	12.500	12.021 (−3.8%)	**12.159 (−2.7**%**)**
**Cr** + PCr	10.000	9.832 (−1.7%)	**10.016 (+0.2**%**)**
**Cho** (GPC + PCh)	3.000	**3.284 (+9.5**%**)**	3.348 (+11.6%)
**mI**/Ins	7.500	5.868 (−21.8%)	**6.014 (−19.8**%**)**
**Glu** + Gln	12.500	12.072 (−3.4%)	**12.311 (−1.5**%**)**
**Lac**	5.000	4.435 (−11.3%)	**4.470 (−10.6**%**)**

Phantom-contained metabolites are bolded in the table and integral-quantified metabolites are shown in the table. Relative error (RE) was calculated by $\mathrm{RE}=({\mathrm{AQC}}_{\mathrm{method}}-{\mathrm{AQC}}_{\mathrm{reference}})/{\mathrm{AQC}}_{\mathrm{reference}}$.

**Table 3:** Influence of SNR with the different kernel sizes in ASSVD.

Methods	WSVD ^1^	ASSVD ^2^ (Kernel Sizes)			
SNR (dB)					
Spectra		3	5	7	9
P1-LA	46	47	48	**49**	49
P1-LB	49	51	**54**	**54**	**54**
P1-LC	44	43	45	**46**	45
P2-LA	48	51	53	**54**	53
P2-LB	52	53	55	55	**56**
P2-LC	35	42	45	**47**	45
P3-LA	42	44	**46**	45	45
P3-LB	49	50	51	51	**52**
P3-LC	42	45	**46**	45	45
P4-LA	33	32	34	36	**37**
P5-LA	34	**37**	**37**	**37**	**37**
P6-LA	32	30	**33**	**33**	32
P7-LA	40	37	39	**40**	**40**
P8-LA	30	**34**	**34**	**34**	33
P9-LB	35	30	33	36	**37**
P9-LC	26	27	28	30	**31**
P10-LB	37	34	35	36	**38**
P10-LC	34	32	35	**38**	37
P11-LB	38	38	39	**40**	40
P11-LC	31	29	32	35	**37**
Average (Increases)	38.9 (0)	39.3 (+0.4)	41.1 (+2.2)	42.1 (+3.2)	**42.2** **(+3.3)**
*p*-value (*t*-test)	**/**	0.2708	**0.001**	**0.000**	**0.000**

^1^ The WSVD is the baseline method. ^2^ The increased SNR compared with the average SNR of WSVD.

## Data Availability

The data presented in this study are available on request from the corresponding author. The data are not publicly available due to the data also forming part of an ongoing study and only being authorized for use in this study. The code demo with simulated data for the test can be found here: https://csrc.xmu.edu.cn/wanqi/ASSVD_poster.htm.
